# Effect of Artesunate on *Leishmania Amazonesis* Induced Neuroinflammation and Nociceptive Behavior in Male Balb/C Mice

**DOI:** 10.3390/ani10040557

**Published:** 2020-03-27

**Authors:** Enrico Gugliandolo, Ernesto Palma, Alessio Filippo Peritore, Rosalba Siracusa, Ramona D’Amico, Roberta Fusco, Patrizia Licata, Rosalia Crupi

**Affiliations:** 1 Department of Chemical, Biological, Pharmaceutical and Environmental Science, University of Messina, 98155 Messina, Italy, aperitore@unime.it (A.F.P.); rsiracusa@unime.it (R.S.); rdamico@unime.it (R.D.); rfusco@unime.it (R.F.); 2 Department of Health Sciences, University of Catanzaro “Magna Graecia”, 88100 Catanzaro, Italy; palma@unicz.it; 3 Department of Veterinary Science, University of Messina, 98168 Messina, Italy

**Keywords:** *Leishmania amazonensis*, inflammation, pain, murine model

## Abstract

**Simple Summary:**

Leishmaniasis is a multisystemic zoonotic disease with several symptoms, and treating this disease is a great challenge for veterinary medicine. Artemisinin derivatives are currently the most widely used drugs for the treatment of malaria, especially for their excellent safety profile and low cost. Artesunate is a more stable derivative of its precursor, artemisin, and has been shown to be a pluripotent agent with different pharmacological actions. In this study, we evaluated the role of neuroinflammation in leishmaniasis and its correlation with pain and sickness behavior, and the anti-inflammatory and neuroprotective effects of artesunate in a murine model of *Leishmania amazonensis* infection in BALB/c mice. The results from this study indicate that artesunate is a good candidate for treatment and/or as an adjuvant in anti- leishmaniasis therapy, and for preventing and alleviating leishmaniasis-induced pain and neuroinflammation.

**Abstract:**

*Background:* Leishmaniasis is a multisystemic zoonotic disease with several symptoms, including neurological disorders. Leishmaniasis is accompanied by an increase in nociceptive behaviors, linked to the presence of a chronic inflammatory state, in both peripheral tissue and the central nervous system. Artesunate is a more stable derivative of its precursor artemisin and has been shown to be a pluripotent agent with different pharmacological actions. *Methods:* In this study, we investigated the effects of artesunate in *Leishmania* *amazonensi-* infected BALB/c mice, evaluating its effectiveness in reducing inflammation, neuroinflammation, and nociceptive and sickness behaviors. *Results:* Our results demonstrate a significant increase in pain sensitivity and sickness behaviors after *L. amazonensis* infection. Moreover, the infection induced a significant increase in inflammatory response at both the paw and spinal cord level. Treatment with artesunate was able to induce a significant decrease in tissue inflammation and neuroinflammation and thus induce a significant decrease in pain sensitivity and sickness behaviors. *Conclusions:* The results from this study indicate that artesunate is a good candidate for treatment and/or as an adjuvant in leishmanicidal therapy, and to prevent and alleviate leishmaniasis-induced pain and neuroinflammation and thereby improve the quality of life of leishmaniasis patients.

## 1. Introduction

Leishmaniasis is a growing public health problem. Leishmania are protozoan parasites with significant diversity, as indicated by the different clinical forms of leishmaniasis, from mild skin lesions, to severe mucosal/cutaneous symptoms, to a lethal visceral form [1]. Leishmania protozoan parasites can affect mammals such as pets; in particular, dogs are the main target of this disease and are considered the main reservoir of the parasite, therefore making the treatment of this pathology in dogs a great challenge for veterinary medicine [2,3]. The principal form of leishmaniasis is localized cutaneous leishmania, with several clinical manifestations in both humans and dogs. This disease represents one of the most frequently occurring painful and inflammatory conditions in humans and animals [4,5]. As reported previously, cutaneous *Leishmania amazonensis* infection induces a significant inflammatory response in infected tissue, with an increase in levels of proinflammatory cytokines such as interleukin (IL)-1β, IL-6, and tumor necrosis factor alpha (TNF-α), and activation of resident and migrating inflammatory cells. This local response has been seen to also induce an increase in inflammatory response in the central nervous system, with increased levels of TNF-α and IL-1β [5], which play key roles in noxious stimuli transmission. This condition of neuroinflammation induced by cutaneous leishmania is responsible for several neurological disorders [6]. The first-line drugs for treating all forms of leishmania are pentavalent antimonial compounds, such as pentostam and glucantime, despite their reported toxicity [7]. These therapies are directed to act against the parasites, and do not act against the possible complications of this infection, such as persistent tissue and neuro- inflammation and concomitant painful sensations. Currently, combination therapy is considered one of the most rational approaches to lower the treatment failure rate and limit the disease’s complications. In canine leishmaniasis, the most frequent treatment protocol chosen is antimoniate meglumine together with allopurinol; the duration of treatment depends on clinical response and individual tolerance [8]. In fact, these therapies also have several side effects, such as xanthinuria, renal mineralization, and urolithiasis in the case of long-term treatment with allopurinol; meglumine antimoniate can be potentially nephrotoxic, and miltefosine can produce gastrointestinal upset [9]. Research groups seek the cure of dogs through new formulations of existing drugs or by associating them [10]

Furthermore, there is a growing interest in veterinary medicine in the search for new strategies for the treatment of leishmania, with particular interest in natural products that can avoid the onset of side effects [11]. Artemisinin is in the sesquiterpene lactone drug family, and is obtained from a Chinese plant, *Artemisia annua*, used for centuries in traditional Chinese medicine [12] and known for its antimalarial properties [13]. Artesunate is a more stable and soluble derivative of artemisinin that has been shown to have different pharmacological actions; it has also been shown to have anti-inflammatory activity [14]. A particular property of artesunate is the capacity to reach and maintain a high concentration in the brain. In fact, artesunate is the most effective drug for the treatment of cerebral malaria, able to reduce inflammation of the brain induced by malaria [15,16]. Previously, it has been seen that artemisinin and artesunate have anti-leishmanial activity in vitro and in vivo while showing limited adverse effects on the host, indicating a higher safety index of the drugs [17]. In this study, we focused on an important property: its action against neuroinflammation [18]. Previously, it has been demonstrated that the use of artesunate in co-therapy with standard therapies is more effective, permitting decreased dosages of both therapies [19]. As shown previously, artesunate has been successfully used in dogs with non-resectable tumors, where in particular it has shown an excellent safety profile for a period of 385 days without the onset of side effects [20]. The aim of this study was to evaluate the potentially protective effect of artesunate treatment at a dose of 30 mg/kg on neuroinflammation, on the painful state induced by *L. amazonensis* cutaneous infection, and, in particular, on induced neuroinflammation and thus on pain and sickness behavior, which are clinical symptoms to consider when treating this condition.

## 2. Materials and Methods

### 2.1. Animals

Male BALB/c mice (6–8 weeks old) were purchased from Envigo (Milan, Italy). The mice were housed in an animal facility with standard conditions (22 ± 1 °C, 12 h light/dark cycle) and with standard rodent food and water ad libitum. Mice were acclimatized to their environment for 1 week. The University of Messina Review Board for the care of animals approved the study (protocol n° 96/2016-PR). Animal care was in accord with Italian (DM 116192) and European Economic Community regulations (OJ of EC L 358/1 12/18/1986) for the protection of experimental animals. The animal study was conducted according to ARRIVE guidelines.

### 2.2. Parasites and Mouse Infections

*L. amazonensis* (WHOM/R/75/Josefa) was cultivated in vitro in Schneider Insect Medium (Sigma-Aldrich) with 10% fetal bovine serum (FBS), 100 U/mL penicillin, and 100 μg/mL streptomycin added. Stationary promastigote forms were transferred to fresh medium at a density of 10^7^ parasites/mL, at 26 °C. BALB/c mice were subcutaneously infected in the right hind footpad with 2 × 10^5^ stationary phase promastigotes of *L. amazonensis*.

### 2.3. Experimental Groups and Treatment

Mice were anesthetized with 5.0% isoflurane in air (Baxter International, Rome, Italy), subcutaneously injected in the right hind paw with 2 × 10^5^ stationary phase promastigotes of *L. amazonensis*, and randomly assigned into the following groups (the experimental protocol was chosen based on what was previously seen [21]):

Control (noninfected) mice (N = 10) were exposed to the same surgical procedure excluding the infection with parasites.

Infected + vehicle mice (N = 10) were subcutaneously infected in the right hind paw with 2 × 10^5^ stationary phase promastigotes of *L. amazonensis*, and 14 days after the infection received a daily intraperitoneal (i.p.) injection of vehicle in which artesunate had been dissolved.

Infected + artesunate mice (N = 10) were subcutaneously infected in the right hind paw with 2 × 10^5^ stationary phase promastigotes of *L. amazonensis*, and after 14 days were treated daily with artesunate (30 m/kg i.p.).

At the end of the experiments, mice were terminally anesthetized with isoflurane (Baxter International, Rome, Italy) and sacrificed by cervical dislocation. The sample size was estimated using a priori power analysis of G*Power software for statistical testing.

### 2.4. Paw Edema

The volume of paw edema was calculated by quantitating the variation in the paw volume using a plethysmometer (model 7140; Ugo Basile). Edema was expressed as increase in paw volume (ml) after *L. amazonensis* injection relative to pre-injection value for each mouse. Results are reported as paw-volume change (ml).

### 2.5. Mechanical Allodynia

Mechanical allodynia was measured by the electronic von Frey test (Bio-EVF4; Bioseb, Vitrolles, France). The device contains a manageable force transducer furnished with a plastic tip. When pressure is applied to the tip, the maximum force applied (in grams) is automatically recorded by the electronic device and exhibited on the screen. The tip was applied to the plantar area of the hind leg, and an upward rising force was exerted until the paw was withdrawn. The withdrawal threshold was defined as the force, in grams, at which the mouse withdrew the paw. The withdrawal was determined three times, and the reported value is the average of the three evaluations.

### 2.6. Thermal Hyperalgesia (Paw Withdrawal Test)

To assess hind paw heat sensitivity, Hargreaves’ test was conducted using a plantar test device (Ugo Basile, Italy). Animals were allowed to freely move within an open-topped transparent plastic box on a glass floor 20 min before the test. A mobile radiant heat source was then placed under the glass floor and focused onto the hind paw. Paw withdrawal latency was measured with a cutoff time of 15 s to prevent tissue damage. The heat stimulation was repeated three times with a 10 min interval to obtain the mean latency of paw withdrawal. Results are expressed as paw withdrawal latency.

### 2.7. Behaviors

#### 2.7.1. Open Field (OF) Test

Locomotor activity was quantified for 5 min in an open field consisting of a white Plexiglas box 59 × 59 cm^2^ with its floor divided into 16 squares. Four squares were defined as the center and the 12 squares along the walls as the periphery. Each mouse was gently placed in the center of the box, and activity was scored as a line crossing when the mouse removed all four paws from one square and entered another. The line crossings and the time spent in the center were counted and scored.

#### 2.7.2. Elevated Plus Maze (EPM)

The elevated plus maze (EPM) apparatus consisted of two open arms (30 × 5 × 0.25 cm^3^) and two enclosed arms (30 × 5 × 15 cm^3^) extending from a common central platform (5 × 5 cm^2^), and the entire apparatus was elevated by supports to a height of 60 cm above floor level. The behavioral model was based on rodents’ aversion to open spaces, which leads to thigmotaxis. Each mouse was placed on the central platform facing an open arm, and its behavior was videotaped with a camera fixed above the EPM. The time spent on the open arms was scored by a researcher blind to the animal treatment. Anxiety was indicated by an increase in the proportion of time spent in the closed arms and a decrease in the proportion of entries into the open arms. The numbers of entries into the open and enclosed arms were scored. The total number of arms was used as a measure of general activity.

### 2.8. Histological Analysis and Myeloperoxidase (MPO) Activity

Tissue sections 7 μm thick were stained with hematoxylin and eosin and then observed by light microscopy coupled with an imaging system (Leica DM2000, Milan, Italy). Histopathological damage was calculated using a single-blinded model from observation of 10 different fields of H&E-stained sections from each animal, as seen previously by Cortes et al. The histopathological-score grading system used to evaluate the degree of histopathological damage was as follows: 0: none; 1: slight infiltration of inflammatory cell; 2: moderate infiltration; 3: severe infiltration [22]. Myeloperoxidase (MPO) activity, an indicator of neutrophil infiltration, was calculated as previously described [23].

### 2.9. Cytokine Determination

Determination of cytokines IL-1β, IL-6, IL-10, and TNF-α was conducted on paw and lumbar spinal cord tissue homogenates by enzyme-linked immunosorbent assay( ELISA )test according to the manufacturer’s protocol (R&D Systems).

### 2.10. Western Blot Analysis

Western blot analysis was performed on the lumbar portion of the spinal cord; samples were homogenized in lysis buffer as described previously [24]. The membranes were probed with specific Abs: anti-glial fibrillary acidic protein (GFAP) (1:1000; Santa Cruz) and anti-ionized calcium binding adaptor molecule 1 (Iba1) (1:1000; Santa Cruz) in 1 × phosphate buffered saline (PBS), 5% w/v nonfat dried milk, and 0.1% Tween-20 at 4 °C overnight. To ascertain that the blots were loaded with equal amounts of proteins, they were also incubated in the presence of the antibody against β-actin protein (cytosolic fraction 1:500; Santa Cruz Biotechnology). Signals were detected with an enhanced chemiluminescence (ECL) detection system reagent according to the manufacturer’s instructions (Thermo, Rockford, IL, USA). The relative expression of protein bands was quantified by densitometry with Bio-Rad ChemiDocTM XRS+ software and standardized to β-actin. Images of blot signals (8 bit/600 dpi resolution) were imported to analysis software (Image Quant TL, v2003). The blot was stripped with glycine 2% and re-probed several times to optimize detection of proteins and visualize other proteins without the need for multiple gels and transfers.

### 2.11. Parasite Quantification

To determine tissue parasite load in the infected group, quantitative PCR (qPCR) for parasite quantification was performed as seen previously [5]. At the end of experimental protocols, a hind paw plantar tissue section was collected, and DNA was extracted using TRIzol reagent following manufacturer’s instructions (Invitrogen, ThermoFisher Carlsbad, CA, USA). The DNA purity was assessed by a spectrophotometer and the ratio (260/280 nm) was between 1.6 and 1.8 for all samples. qPCR was performed using Platinum SYBR Green qPCR SuperMix UDG with ROX reagent (Invitrogen, ThermoFisher) with 100 ng total genomic DNA (gDNA). Parasite quantification was performed with Leishmania specific primers JW11 (forward, 50 -CCTATTTTACACCAACCCCCAGT-30) and JW12 (reverse, 50 -GGGTAGGGGCGTTCTGCGAAA-30). The results were presented as parasite DNA expression, using b-actin as a reference gene to normalize data.

### 2.12. Materials

Artesunate was obtained from Santa Cruz Biotechnology. All compounds used in this study, except where otherwise specified, were purchased from Sigma-Aldrich Company Ltd. Artesunate powder was dissolved beforehand in dimethyl sulfoxide (DMSO), to obtain a final solution of 10% DMSO, which we have seen to be the minimum to prevent salting out. All solutions used for in vivo administration were made using non-pyrogenic saline (0.9% wt/vol NaCl; Baxter Healthcare Ltd., Thetford, UK).

### 2.13. Statistical Analysis

The data were analyzed by one-way ANOVA followed by a Bonferroni post hoc test for multiple comparisons. A *p*-value of less than 0.05 was considered significant. Data are expressed as mean ± SEM from N = 10 mice for each group.

## 3. Results

### 3.1. Effect of Artesunate on L. amazonensis Induced Paw Edema and Pain Behavior

**Figure 1:**
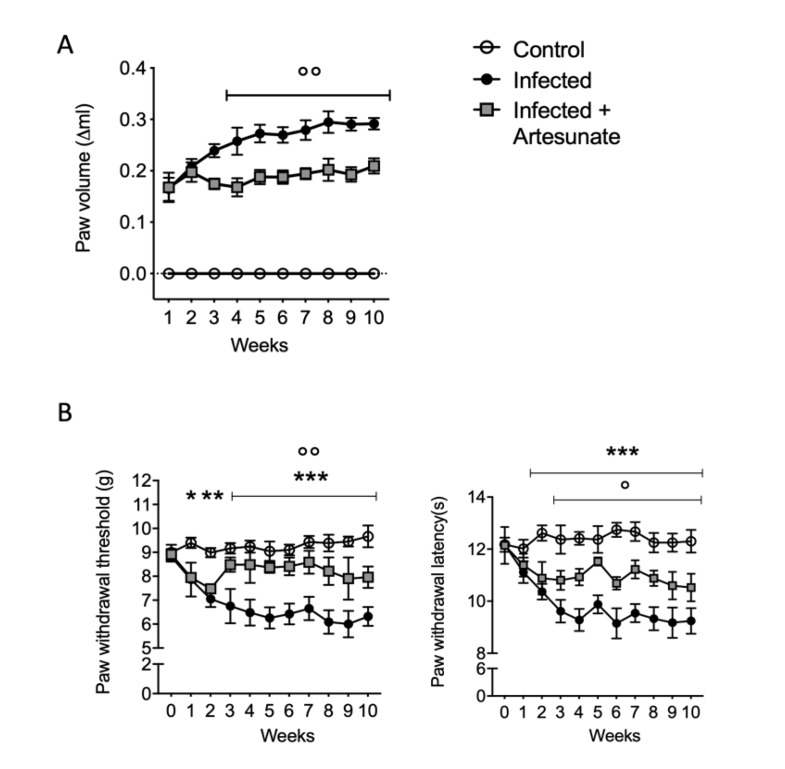
(**A**) Time course of paw volume after *L. amazonensis* infection; (**B**) time course of mechanical allodynia as paw withdrawal threshold (g) and thermal hyperalgesia as paw withdrawal latency. Data are expressed as mean ± SEM from N = 10 mice for each group. *** *p <* 0.001 vs. control group; ** *p <* 0.01 vs. control group; * *p <* 0.05 vs. control group; °° *p <* 0.01 vs. infected group; ° *p <* 0.05 vs. infected group.

As shown in Figure 1A, after subcutaneous infection with *L. amazonensis* in the right hind paw, we observed an increase in paw volume during the experimental protocol in the infected group. The treatment with artesunate started two weeks after the infection, and starting from the fourth week, compared to the infected group, the treated group showed significantly reduced paw volume. Leishmanial skin lesions are often associated with painful states [4,25,26]. Therefore, we evaluated the effects of artesunate on mechanical allodynia, measured by the electronic von Frey test, and thermal hyperalgesia, by the paw withdrawal test. As shown in Figure 1B, we observed a significantly decreased threshold for both mechanical and thermal stimuli in the *L. amazonensis*-infected group. In the artesunate group, after seven days of treatment, we observed a significantly increased threshold for mechanical and thermal stimuli, and this was more evident for the mechanical stimuli.

### 3.2. Effect of Artesunate on L. amazonensis-Induced Sickness Behavior

**Figure 2:**
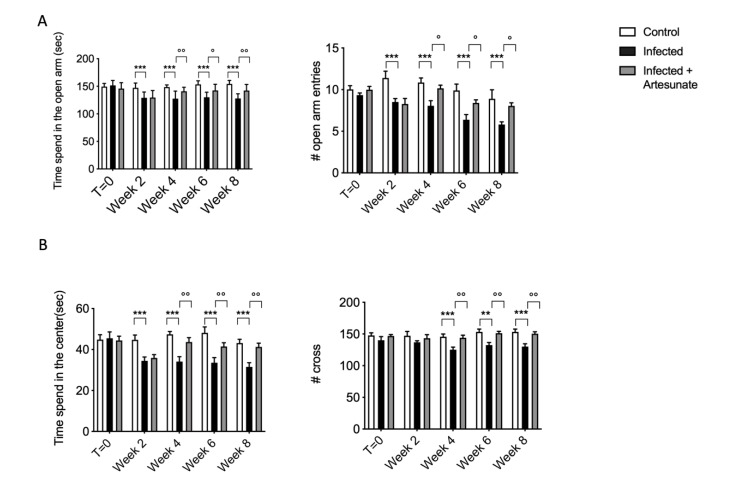
(**A**) An elevated plus maze test showed a significant decrease in time spent on open arms and number of open arm entries for the infected group compared to the control group. Treatment with artesunate significantly increased time spent on open arms and number of open arm entries compared to infected group. (**B**) An open field test showed a significant decrease in time spent in the center and the number of lines crossed in the infected group compared to control group. Treatment with artesunate significantly decreased the time spent in the center and the number of lines crossed compared to infected group. Data are expressed as mean ± SEM from N = 10 mice for each group. *** *p <* 0.001 vs. control group; ** *p <* 0.01 vs. control group; °° *p <* 0.01 vs. infected group; ° *p <* 0.05 vs. infected group.

Evidence suggests a link between infection/inflammation and sickness behavior in humans and especially in animals. In mice this kind of behavior can affect the performance in tests used to assess anxiety and depression. In this study we evaluated mouse behavior by the elevated plus maze (EPM) and the open field (OF). The results from the EPM test (Figure 2A) show that, after *L. amazonensis* infection, in the infected group there was a significant decrease in open arm entries and time spent on open arms. This is generally related to anxiety-like behavior, and the number of open arm entries indicates a decrease in general activity. Daily treatment with artesunate in part reversed the effect seen in the infected group. In the OF test, we also observed a significant reduction, in the infected group, in the time spent in the center and in line crossing during the observation. Treatment with artesunate starting from two weeks after infection in part prevented this altered behavior. It has been well recognized and demonstrated that in animals this kind of behavior is induced by bacterial infection or, in general, as a systemic or neuro-inflammatory response through activation of immune-neural mechanisms and modulated by cytokine levels [27].

### 3.3. Effect of Artesunate on L. amazonensis-Induced MPO and Histological Damage

**Figure 3:**
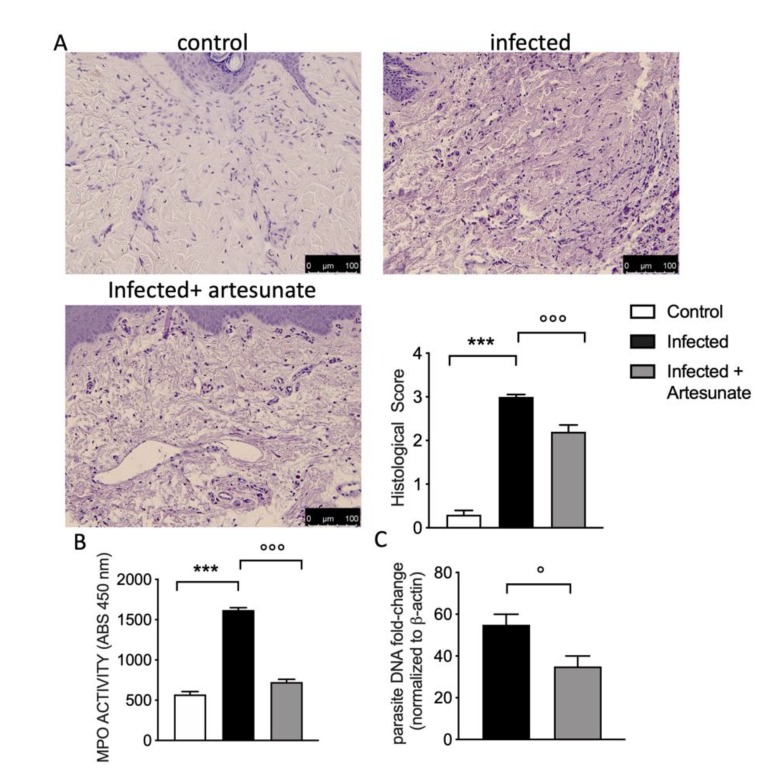
(**A**) Hematoxylin and eosin (H&E) staining and histological score graph of infected paw tissues from control, infected, and artesunate groups. (**B**) Myeloperoxidase (MPO) assay highlights the presence of inflammatory cells. *L. amazonensis* infection induced a significant increase in MPO, and the artesunate group showed a significant reduction in MPO compared to the infected group. (**C**) qPCR for parasitism in paw tissue evaluation. Data are expressed as mean ± SEM from N = 10 mice for each group. *** *p <* 0.001 vs. control group; ° *p <* 0.05 vs. infected group; °°° *p <* 0.001 vs. infected group.

According to paw edema results, ten weeks after infection, histological analysis of the infected right hind paw tissues (Figure 3A) shows significant histological damage, with the presence of edema and inflammatory cell recruitment, compared to the infected group. Daily artesunate treatment started two weeks after the infection was able to partially prevent these histological alterations. The presence of inflammatory cells was also seen by MPO assay, as shown in Figure 3B. Ten weeks after *L. amazonensis* infection, there was a significant increase in MPO, and the group treated with artesunate showed a significant reduction in MPO compared to the control group. *L. amazonensis* parasitism in paw tissue was evaluated by qPCR ten weeks after infection, as shown in Figure 3C.

### 3.4. Effect of Artesunate on L. amazonensis-Induced IL-1β, IL-6, and TNF-α in Paw Tissue

**Figure 4:**
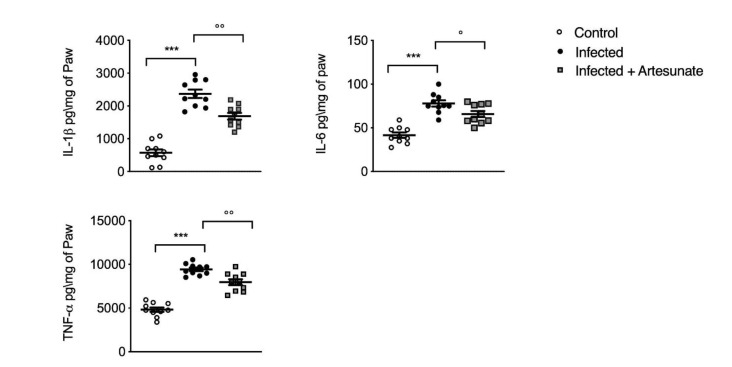
ELISA assay for interleukin (IL)-1β, IL-6, and tumor necrosis factor alpha (TNF-α) in paw tissue. Data are expressed as mean ± SEM from N = 10 mice for each group. *** *p <* 0.001 vs. control group; °° *p <* 0.01 vs. infected group; ° *p <* 0.05 vs. infected group.

As indicated from H&E staining and MPO assay, the inflammatory process was confirmed by the presence of principal proinflammatory cytokines in paw tissue by ELISA test. In particular, as shown in Figure 4, the infected group showed significantly increased levels of induced IL-1β, IL-6, and TNF-α 10 weeks after *L. amazonensis* injection in the right hind paw. Daily treatment with artesunate, started two weeks after the infection, was able to significantly reduce IL-1β, IL-6, and TNF-α levels in paw tissue.

### 3.5. Effect of Artesunate on L. amazonensis-Induced IL-1β, IL-10, and TNF-α in Spinal Cord Tissue

**Figure 5:**
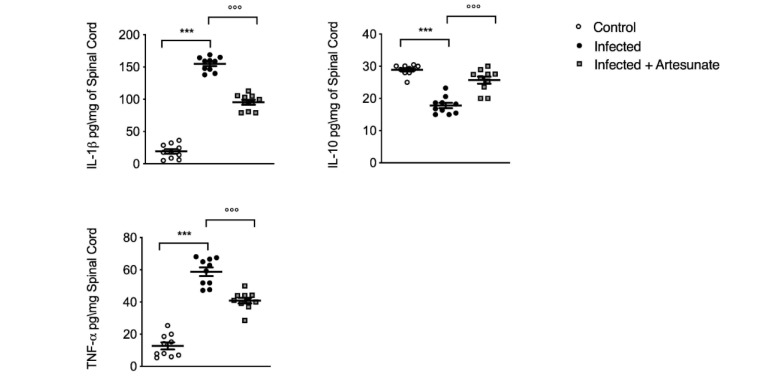
ELISA assay for IL-1β, IL-10, and TNF-α in lumbar spinal cord tissue. Data are expressed as mean ± SEM from N = 10 mice for each group. *** *p <* 0.001 vs. control group; °°° *p <* 0.001 vs. infected group.

We then we evaluated the presence of inflammation at the neuronal level, a key event in pain sensibilization. As shown in Figure 5, for IL-1β and TNF-α, we found significantly increased levels in spinal cord tissue in the infected group ten weeks after infection, and daily artesunate treatment, started two weeks after the infection, significantly reduced the levels of IL-1β and TNF-α. Interestingly, the presence of these cytokines in the spinal cord is a key factor in pain sensibilization. We also evaluated the level of IL-10, which has a role in the regulation of inflammatory response through an “anti-inflammatory” action. In this case, we found in the infected group a significant reduction in IL-10 levels compared to the control group. Treatment with artesunate, compared to the infected group, showed a significant increase in IL-10 levels.

### 3.6. Effect of Artesunate on L. amazonensis-Induced Astrocyte and Microglia Activation

**Figure 6:**
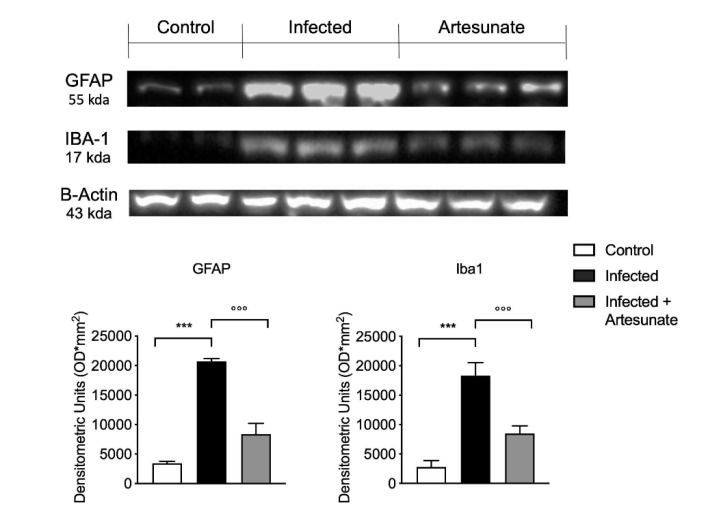
Western blot analysis of glial fibrillary acidic protein (GFAP) and ionized calcium binding adaptor molecule 1 (Iba1) expression in spinal cord tissue. Data are expressed as mean ± SEM from N = 10 mice for each group. *** *p <* 0.001 vs. control group; °°° *p <* 0.001 vs. infected group.

Astrocyte and microglia activation is critical in neuroinflammation and thus in the neuropathic pain state. A marker of astrocyte and microglia activation is increased expression of glial fibrillary acidic protein (GFAP) and ionized calcium-binding adaptor molecule 1 (Iba1). As shown in Figure 6, we observed a significant increase in expression of GFAP and Iba1 in the infected group 10 weeks after the infection; in comparison, the artesunate-treated group, with treatment started two weeks after the infection, showed a significant reduction of GFAP and Iba1 expression in spinal cord tissue.

## 4. Discussion

The main idea of this study was to evaluate the role of artesunate treatment in inflammation, neuroinflammation and sickness behavior in *L. amazonensis* infection, and thus to evaluate artesunate as a possible strategy for the management of pain and neuroinflammation in leishmaniasis. The term leishmaniasis includes multiple clinical syndromes, including cutaneous, mucosal, and visceral forms, resulting from a protozoan parasitic infection of the genus Leishmania. Leishmaniasis is a growing public health disease that infects wild and domestic mammals, including humans. In particular leishmaniasis is considered a great challenge in veterinary medicine; in fact, the domestic dog is the only reservoir host of major veterinary importance, and also the management of this pathology in dogs is a great challenge to improve their quality of life [2,3]

Tissue damage and disease are caused by an excessive and prolonged host immune response due to the presence of parasites, characterized by an important and diffuse infiltration of inflammatory cells (including macrophages, lymphocytes, neutrophils, mast cells, and plasma cells) [28]. Cutaneous leishmaniasis is a worldwide sanitary problem for both humans and animals, in particular dogs [29]. An increasing amount of evidence indicates that Leishmania infection induces hyperalgesia in domestic animals such as dogs [25,26].

We chose a model of cutaneous leishmania induced by subcutaneous injection of *L. amazonensis* to assess the effect of infection in the development of neuroinflammation and related pain, and artesunate as a potential pharmacological approach. Artesunate is a derivative of artemisin, from a Chinese plant, *Artemisia annua*, used for centuries in traditional Chinese medicine. Artesunate is a water-soluble, semi-synthetic derivate of artemisin. The World Health Organization (WHO) recommends artesunate as the drug of choice for severe malaria in adults and for the cerebral form of malaria [30,31]. Interestingly, treatment with artesunate has also been seen to reduce the inflammation associated with this disease [15,32]. Artesunate is characterized by the ability to reach and maintain a high concentration in the brain, thus it is an interesting drug for the treatment of the central nervous system and neurological disorders associated with neuroinflammation [16,33]. Artesunate has also been seen to have anti-leishmanial activity in vitro and in vivo [19] and in *Babesia equi* infection [34]. Even though there are important perspectives on the use of artesunate in therapy against parasitic diseases such as leishmania for both humans and dogs [17,35], contrary to human medicine in veterinary medicine, clinical studies on the treatment of canine leishmaniasis are few and certainly new studies will be needed to clarify the use of this compound in clinical practice. Several studies have shown the use of artesunate in dogs, showing its anti-schistosomal efficacy [36,37], but, above all, several studies have highlighted its good therapeutic profile in dogs, given the low incidence of side effects [20]. Even though there are important perspectives on the use of artesunate in therapy against parasitic diseases such as leishmania for both humans and dogs [17,35], in this study we focused on an important property, its action against neuroinflammation [18], and thus its action again inflammatory pain induced by *L. amazonensis* infection. It has previously been demonstrated that the use of artesunate in co-therapy with standard therapies is more effective than its use in monotherapy, permitting decreased dosages of both therapies [19]. Our results show that, after injection of *L. amazonensis* in the right hind paw, there was a significant increase in paw volume. When compared with the infected group, the artesunate group, treated daily with a dose of 30mg/kg, starting two weeks after infection, had significantly reduced paw volume.

Leishmanial skin lesions are often associated with painful states [4,38]; thus, we evaluated the effects of artesunate on mechanical allodynia and thermal hyperalgesia. Our results show a significant increase in nociceptive sensibilization to both mechanical and thermal stimuli after *L. amazonensis* infection. The group treated with artesunate showed a reduction in this hypersensitivity compared to the infected group. H&E staining in the infected group revealed important tissue damage with the presence of edema and abundant inflammatory cells, also confirmed by the high MPO levels observed in the infected group. Compared to the infected group, the group treated with artesunate showed a significantly lower level of histological alteration, with a reduction of inflammatory cell infiltration and thus a lower MPO level. This hyper-sensibility has been seen to be related to the inflammatory response induced by protozoic infection in peripheral tissue, such as the infected paw, and in the central nervous system (CNS), with the involvement of resident immune cells in the CNS and in the periphery [21]. These processes are finely regulated by cytokines, especially the principal inflammatory cytokines IL-1β and TNF-α [39]. These molecules can both activate and sensitize the spinal cord neurons that transmit peripheral nociceptive information to the brain.

It has been seen that inflammatory response, neuroinflammation, and pain conditions in animals are related to sickness behavior [40,41]. High and prolonged production of proinflammatory cytokines can cause damage to tissue and to the CNS, producing severe behavioral alterations, such as depression and anxiety [42]. For this reason, in our study, we evaluated alterations in behavior after *L. amazonensis* infection and the potential therapeutic effect of artesunate. In rodents, sickness behavior can be assessed by classical behavioral analysis, which is useful for assessing anxiety-like behaviors. In this study, we evaluated mouse behavior by elevated plus maze (EPM) and open field (OF) tests. Tests showed that after *L. amazonensis* infection, in the infected group, there was a significant decrease in open arm entries and time spent on open arms, generally related to anxiety-like behavior, and the number of open arm entries, indicating a decrease in general activity. Daily treatment with artesunate in part reversed the effects seen in the infected group. Additionally, in the OF test, in the infected group we observed a significant reduction in time spent in the center and in line crossing during the observation. Treatment with artesunate starting from two weeks after infection in part prevented this altered behavior.

We then confirmed the inflammatory response in peripheral tissue and in CNS and evaluated the effect of artesunate on these processes. In paw tissue from infected mice after infection, we found a significantly increased level of the principal proinflammatory cytokines IL-1β, IL-6, and TNF-α, while treatment with artesunate significantly reduced the levels of these cytokines. The presence of high levels of these cytokines is known to be a fundamental step in noxious stimulation in CNS. Next, we evaluated the levels of proinflammatory IL-1β and TNF-α and the anti-inflammatory cytokine IL-10 in spinal cord. In spinal cord from infected animals, we found a significant increase in IL-1β and TNF-α levels that was significantly reduced by artesunate treatment. The presence of high levels of IL-1β and TNF-α in spinal cord is closely related to pain hypersensitivity [43]. For IL-10, which has a fundamental role in regulating inflammatory response, in particular, it has been seen that it has an important role in inhibiting neuroinflammation, in part by modulating microglia and astrocyte activation [44]. In spinal cord from infected animals, compared to the control group, we found significantly decreased IL-10 levels, which were significantly increased by artesunate treatment. It has been seen that *L. amazonensis* skin infection produces chronic pain by central mechanisms involving spinal cord astrocyte and microglia activation and production of cytokines and chemokines, thus leading to infection-induced hyperalgesia, neuroinflammation [45], and behavior alteration [6]. Microglia and astrocyte activation were analyzed by the expression of Iba-1, one of the most used microglia/macrophage activation markers, and of GFAP, for astrocytes. Our results show that 10 weeks after infection, in the infected group, there was significant increase in Iba-1 and GFAP expression in spinal cord tissue. Artesunate treatment significantly reduced Iba-1 and GFAP expression, according to the trend seen for IL-10.

## 5. Conclusions

In conclusion, the outcome from this study provides evidence that cutaneous leishmaniasis is a complex disease that involves the CNS and suggests the use of artesunate as a possible pharmacological strategy for the management of this condition. In fact, our results show peripheral tissue inflammation, spinal cord neuroinflammation and resident immune cell activation induced by *L. amazonensis* infection, events that are related to important pain hyper-sensibilization and induced sickness behavior. These events should be considered in modern pharmacological protocols for leishmaniasis treatment, which aim to achieve a reduction in infection burden and prevent related complications, especially for the long duration of this disease and thus of therapy. The results from this study indicate that artesunate is a good candidate for treatment and/or as an adjuvant in leishmanicidal therapy, to prevent and alleviate leishmaniasis-induced pain and neuroinflammation and thereby improve the quality of life of leishmaniasis patients.
